# Cyclic vomiting syndrome in children: a nationwide survey of current practice on behalf of the Italian Society of Pediatric Gastroenterology, Hepatology and Nutrition (SIGENP) and Italian Society of Pediatric Neurology (SINP)

**DOI:** 10.1186/s13052-022-01346-y

**Published:** 2022-08-30

**Authors:** Sara Isoldi, Giovanni Di Nardo, Saverio Mallardo, Pasquale Parisi, Umberto Raucci, Renato Tambucci, Paolo Quitadamo, Silvia Salvatore, Enrico Felici, Fabio Cisarò, Licia Pensabene, Claudia Banzato, Caterina Strisciuglio, Claudio Romano, Patrizia Fusco, Francesca Rigotti, Naire Sansotta, Silvia Caimmi, Salvatore Savasta, Giovanna Zuin, Marina Di Stefano, Silvia Provera, Angelo Campanozzi, Paolo Rossi, Simona Gatti, Mara Corpino, Patrizia Alvisi, Stefano Martelossi, Agnese Suppiej, Paolo Gandullia, Alberto Verrotti, Gianluca Terrin, Caterina Pacenza, Fabiola Fornaroli, Donatella Comito, Stefano D’Arrigo, Pasquale Striano, Federico Raviglione, Marco Carotenuto, Alessandro Orsini, Vincenzo Belcastro, Giovanna Di Corcia, Vincenzo Raieli, Michela Ada Noris Ferilli, Claudia Ruscitto, Elisabetta Spadoni, Salvatore Grosso, Renato D’Alonzo, Amanda Papa, Piero Pavone, Mariaclaudia Meli, Mario Velardita, Martina Mainetti, Nicola Vanacore, Osvaldo Borrelli

**Affiliations:** 1 Maternal and Child Health Department, Santa Maria Goretti Hospital, Sapienza-University of Rome, Latina, Italy; 2 Department of Neuroscience, Mental Health and Sense Organs (NESMOS), Faculty of Medicine & Psychology, Sant’Andrea Hospital, Sapienza University of Rome, 00189 Rome, Lazio Italy; 3 Pediatric Emergency Department, Bambino Gesù Children’s Hospital, Institute for Research, Hospitalization and Health Care (IRCCS), Rome, Italy; 4 Digestive Endoscopy and Surgery Unit, Bambino Gesù Children’s Hospital, Institute for Research, Hospitalization and Health Care (IRCCS), Rome, Italy; 5 Department of Pediatrics, A.O.R.N. Santobono-Pausilipon, Naples, Italy; 6 Pediatric Department, Ospedale “F. Del Ponte”, University of Insubria, Varese, Italy; 7 Unit of Pediatrics, The Children Hospital, Azienda Ospedaliera SS Antonio e Biagio e Cesare Arrigo, Alessandria, Italy; 8 Pediatric Gastroenterology Unit, Regina Margherita Children’s Hospital, Azienda Ospedaliera-Universitaria, Città della Salute e della Scienza di Torino, Turin, Italy; 9 Pediatric Unit, Department of Medical and Surgical Sciences, University “Magna Graecia” of Catanzaro, Catanzaro, Italy; 10 Department of Surgical Sciences, Dentistry, Gynecology and Pediatrics, Pediatric Division, University of Verona, Verona, Italy; 11 Department of Woman, Child, General and Specialistic Surgery, University of Campania “Luigi Vanvitelli”, Naples, Italy; 12 Pediatric Gastroenterology Unit, Department of Human Pathology in Adulthood and Childhood “G. Barresi”, University of Messina, Messina, Italy; 13 Pediatric Unit, Montis Regalis Hospital, Mondovì, Cuneo, Italy; 14 Department of Pediatrics, Bolzano Hospital, Bolzano, Italy; 15 Paediatric Hepatology, Gastroenterology and Transplantation, Hospital Papa Giovanni XXIII, Bergamo, Italy; 16 Pediatric Clinic, Foundation IRCCS Policlinico San Matteo, University of Pavia, Pavia, Italy; 17 Pediatric Department, University of Milano Bicocca, FMBBM, San Gerardo Hospital, Monza, Italy; 18 Department of Pediatrics, IRCCS San Raffaele Scientific Institute, Milan, Italy; 19 Department of Pediatrics, IRCCS Sacro Cuore Don Calabria Hospital, Negrar, Verona, Italy; 20 Department of Medical and Surgical Sciences, Pediatric Unit, University of Foggia, Foggia, Italy; 21 Department of Pediatrics, Pediatric Gastroenterology and Liver Unit, Sapienza University of Rome, Rome, Italy; 22 Department of Pediatrics, Università Politecnica delle Marche, Ancona, Italy; 23 Gastroenterologia Pediatrica, Clinica Pediatrica e Malattie Rare, Ospedale Pediatrico Microcitemico A. Cao, ARNAS Brotzu, Cagliari, Italy; 24 Department of Paediatrics, Ospedale Maggiore, Azienda USL, Bologna, Italy; 25 Pediatric Unit, Ca’ Foncello’s Hospital, Treviso, Italy; 26 Pediatric Section, Department of Medical Sciences, University of Ferrara, Ferrara, Italy; 27 UOC Gastroenterologia, IRCCS Istituto Giannina Gaslini, Genoa, Italy; 28 Department of Pediatrics, University of Perugia, Perugia, Italy; 29 Department of Maternal and Child Health, Policlinico Umberto I, Sapienza University of Rome, Rome, Italy; 30 Department of Pediatrics, San Giovanni di Dio Hospital, Crotone, Italy; 31 Gastroenterology and Endoscopy Unit, Department of Medicine and Surgery, University of Parma, Parma, Italy; 32 Ospedale Santa Croce, Moncalieri, Italy; 33 Developmental Neurology Division, Fondazione IRCCS Istituto Neurologico Carlo Besta, Milan, Italy; 34 Pediatric Neurology Unit, IRCCS Istituto Giannina Gaslini, Genoa, Italy; 35 Hospital Neuropsychiatry Service, ASST Rhodense, Rho, Milan, Italy; 36 Clinic of Child and Adolescent Neuropsychiatry, Department of Mental Health and Physical and Preventive Medicine, Università degli Studi della Campania “Luigi Vanvitelli”, Caserta, Italy; 37 Paediatric Neurology Section, Paediatric Department, Pisa University Hospital, University of Pisa, Pisa, Italy; 38 Neurology Unit, Maggiore Hospital, Lodi, Italy; 39 Pediatric Department, “Augusto Murri” Hospital, Fermo, Italy; 40 Child Neuropsychiatry Unit - ISMEP- ARNAS CIVICO, Palermo, Italy; 41 Department of Neuroscience, Paediatric Headache Center, Bambino Gesù Children Hospital, IRCCS, Rome, Italy; 42 Child Neurology and Psychiatry Unit, Systems Medicine Department, Tor Vergata University Hospital of Rome, Rome, Italy; 43 Pediatric Department, Azienda Ospedaliera Nord-Ovest of Tuscany, San Luca Hospital of Lucca, Lucca, Italy; 44 Molecular Medicine and Development, University of Siena, Siena, Italy; 45 Pediatric and Neonatological Unit, Maternal and Child Department, Nuovo Ospedale San Giovanni Battista, Foligno, Italy; 46 Infantile Neuropsychiatry Departement Maggiore della Carità University Hospital, Novara, Italy; 47 Section of Pediatrics and Child Neuropsychiatry, Department of Clinical and Experimental Medicine, University of Catania, Catania, Italy; 48 Azienda Policlinico, “Rodolico-San Marco Hospital”, University of Catania, Catania, Italy; 49 Department of Pediatrics, Hospital “Salvatore e Saverio Gravina”, Caltagirone, Italy; 50 Department of Pediatrics, Santa Maria delle Croci Hospital, Ravenna, Italy; 51 National Centre for Epidemiology, Surveillance and Health Promotion, National Institute of Health, Rome, Italy; 52 Division of Neurogastroenterology and Motility, Department of Pediatric Gastroenterology, University College London (UCL) Institute of Child Health and Great Ormond Street Hospital, London, UK

**Keywords:** Cyclic vomiting, Management, Outcomes, Pediatric

## Abstract

**Background:**

Cyclic Vomiting Syndrome (CVS) is a rare functional gastrointestinal disorder, which has a considerable burden on quality of life of both children and their family. Aim of the study was to evaluate the diagnostic modalities and therapeutic approach to CVS among Italian tertiary care centers and the differences according to subspecialties, as well as to explore whether potential predictive factors associated with either a poor outcome or a response to a specific treatment.

**Methods:**

Cross-sectional multicenter web-based survey involving members of the Italian Society of Pediatric Gastroenterology, Hepatology and Nutrition (SIGENP) and Italian Society of Pediatric Neurology (SINP).

**Results:**

A total of 67 responses were received and analyzed. Most of the respondent units cared for less than 20 patients. More than half of the patients were referred after 3 to 5 episodes, and a quarter after 5 attacks. We report different diagnostic approaches among Italian clinicians, which was particularly evident when comparing gastroenterologists and neurologists. Moreover, our survey demonstrated a predilection of certain drugs during emetic phase according to specific clinic, which reflects the cultural background of physicians.

**Conclusion:**

In conclusion, our survey highlights poor consensus amongst clinicians in our country in the diagnosis and the management of children with CVS, raising the need for a national consensus guideline in order to standardize the practice.

**Supplementary Information:**

The online version contains supplementary material available at 10.1186/s13052-022-01346-y.

## Background

Cyclic Vomiting Syndrome (CVS) is a rare functional gastrointestinal (GI) disorder characterized by stereotypical and repetitive episodes of vomiting and intense nausea lasting between few hours and several days [1–3]. The episodes are interspersed with periods of variable duration during which the patients return to their baseline health [1–3]. The incidence of CVS is estimated at 3.2 per 100,000 subjects, whilst the prevalence ranges between 1.9 and 2.3% [4–7]; however, the latter could be an underestimation due to either under-ascertainment or under-diagnosis of the disorder [8].

CVS has a considerable burden on quality of life of both children and their family due to the frequent hospital admissions and its repercussion on both social and academic functioning ability [9, 10]. Moreover, the syndrome is associated with significant health care costs, including regular outpatient assessment, emergency department visits, prolonged hospital admission and use of diagnostic investigation, advocating the need of a cost-effectiveness management for limiting its financial impact on healthcare system [11].

Although our understanding of the pathogenesis of childhood CVS is still limited, several mechanisms have been postulated, including hypothalamic-pituitary-adrenal activation, autonomic dysfunction, polymorphisms of mitochondrial DNA, genetic abnormalities, neuronal hyperexcitability, and gastric dysmotility [12]. Noteworthy, the high prevalence of both personal and family history of migraine, the effectiveness of anti-migraine therapy, the presence of mitochondrial DNA polymorphisms and the frequent developmental progression from CVS to migraine later in life suggest a common pathophysiologic process and, in turn, to consider CVS as a migraine-related or migraine-equivalent disorder [13–16]. Hence, it is not uncommon that CVS is managed by both pediatric gastroenterologists and pediatric neurologists.

The clinical history suffices to diagnose CVS and the vast majority of children fulfilling the current criteria are generally labeled as having an idiopathic form. However, in a subgroup of children either life-threatening or treatable disorders need to be excluded. As an indiscriminate diagnostic approach is costly, time-consuming and invasive, it is highly recommended that the main organic causes are excluded through pinpoint investigations according to clinical suspicion [1]. Moreover, the current therapeutic approach aims at identifying and avoiding individual triggers, aborting the acute phase and either preventing the emetic cycles or decreasing their frequency and intensity [17, 18]. However, given the lack of evidence regarding the effectiveness of any specific treatment, CVS management is still primarily based on clinical expertise and expert-opinions. Therefore, the decision-making approach might be significantly affected by the cultural background of physicians dealing with CVS children.

The present study aimed to evaluate the diagnostic modalities and therapeutic approach to CVS among Italian tertiary care centers and the differences according to subspecialties, as well as to explore whether potential predictive factors associated with either a poor outcome or a response to a specific treatment.

## Methods

This cross-sectional multicenter web-based survey was conducted between October 2019 and September 2020. A questionnaire was developed by the core group of researchers (GDN, UR, PP, SM, RT, PQ and OB) with the aim to assess practices in tertiary Italian centers treating children with CVS. The concept for the survey was developed in July 2019, during the Neurogastroenterology and Motility working group meeting of the Italian Society of Pediatric Gastroenterology, Hepatology and Nutrition (SIGENP), which also saw the attendance of delegates of the Italian Society of Pediatric Neurology (SINP). After discussion with the working group members and representative from the leading pediatric gastroenterology and neurology centers in Italy, the authors identified the main controversies around the epidemiology, diagnosis and management of CVS, which were subsequently included in the questionnaire. The survey was initially created by GDN, SM, RT and PP, and then critically reviewed and edited through 2 rounds by PQ and OB. The final version of the questionnaire was sent electronically to all the members of the SIGENP and SINP. Reminders were sent every 3 months via direct e-mail to ensure that the survey was completed and being representative of major centers scattered throughout the Italian national territory dealing with CVS patients. The questionnaire included 14 multiple-choice questions and 7 open-ended questions, with the former made compulsory in order to achieve the successful completion of the survey. The questionnaire is presented in the supplementary material (Supplementary Table 1). The survey did not require the disclosure of the responder’s identity, but only the name of the hospital and the city. The survey was distributed in accordance with standard survey distribution policy. The study protocol conforms to the ethical guidelines of the 1975 Declaration of Helsinki (6th revision, 2008). The main focus was to determine current practices in relation to the diagnosis and management of patients with CVS in centers across Italy.

### Statistical analysis

The results were evaluated by means of simple descriptive analysis. Kruskal-Wallis one-way analysis of variance by ranks, followed by Chi-square test. Likelihood Ratio test (LR) was applied to test out the significance of association among the variables. The level of significance was set as 0.05, therefore, if any *p*-value observed less than 0.05 was considered as statistical significance. Statistical analysis was performed by running the Software Statistical Package for Social Sciences version 21 (SPSS Inc., Chicago, Illinois USA).

## Results

**Fig. 1:**
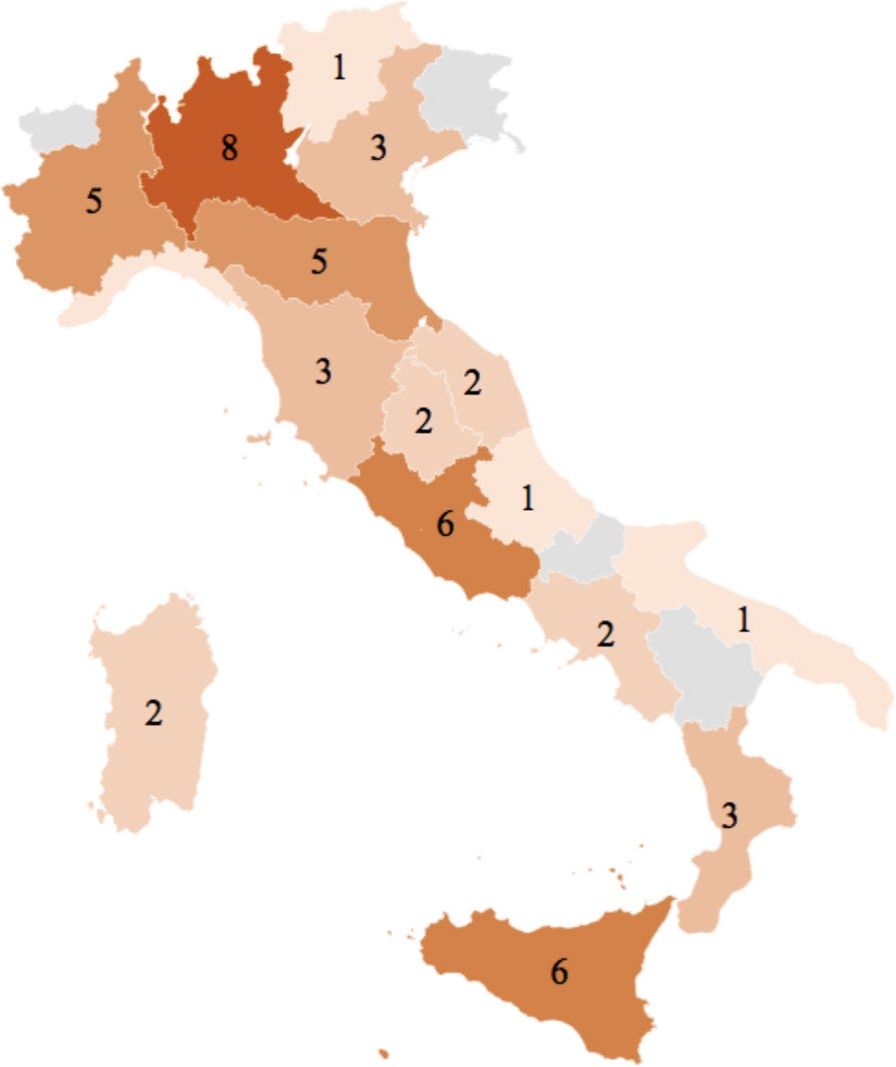
Distribution of participating centers across Italy

A total of 67 responses belonging to different clinics from 51 centers were received and analyzed. Figure 1 provides the distribution of participating centers across Italy. The majority of respondents were from the North of Italy (26, 38.8%), followed by the South (23, 34.3%, including the isles) and the Center (18, 26.9%). The number of patients with CVS cared among clinics was > 100 patients in 1 (1.5%), between 50 and 100 patients in 3 (4.5%), between 20 and 49 patients in 4 (6%), between 10 and 19 patients in 22 (32.8%) and less than 10 patients in 37 (55.2%).

**Fig. 2:**
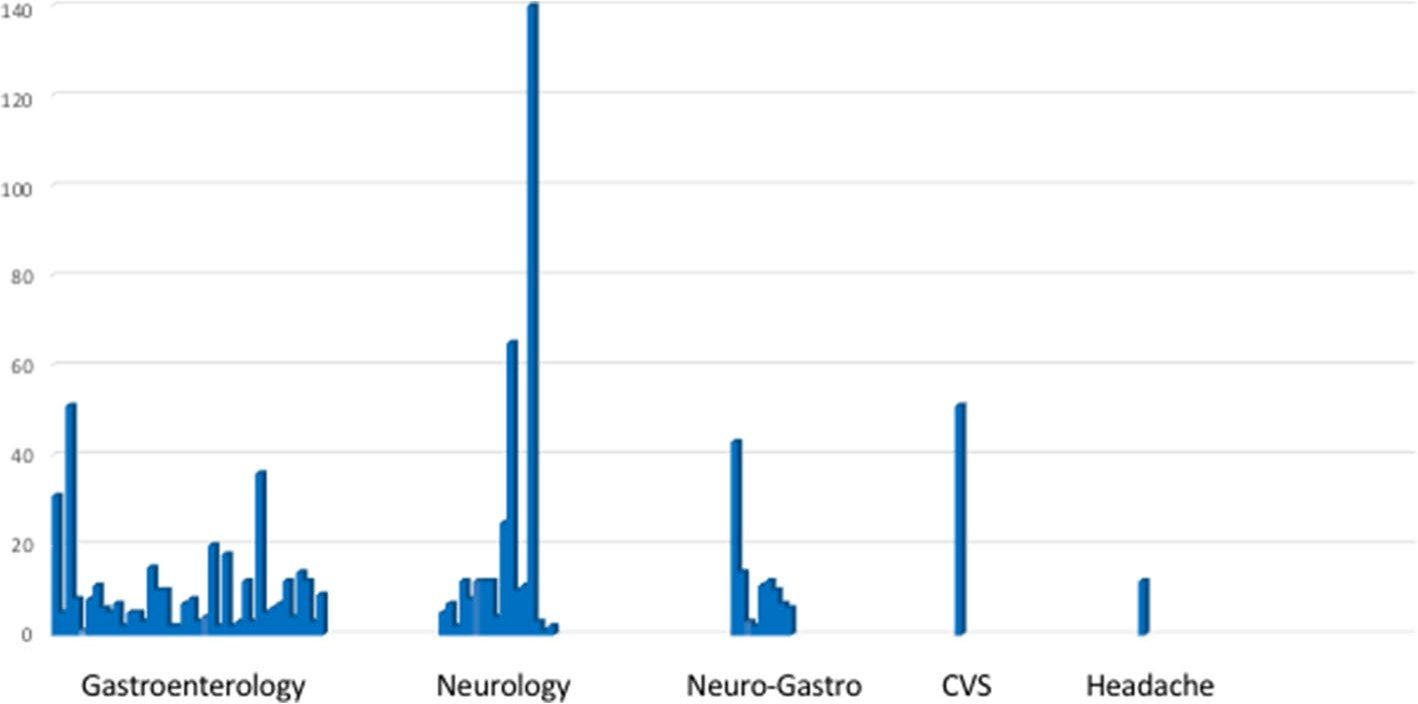
Number patients with cyclic vomiting syndrome cared per specific clinic

Patients were followed-up in the gastroenterology outpatient clinic at 41 centers (61.2%), in the neurology outpatient clinic at 15 centers (22.4%), in the combined neuro-gastroenterology outpatient clinic at 9 centers (13.4%), in the headache outpatient clinic at 1 center (1.5%) and in a specific CVS outpatient clinic at 1 center (1.5%). Figure 2 depict the number of patients cared per specific clinic. No significant difference was found regarding number of patients cared according to specific clinic (*p* = 0.386, ns).

### Diagnostic criteria

**Fig. 3:**
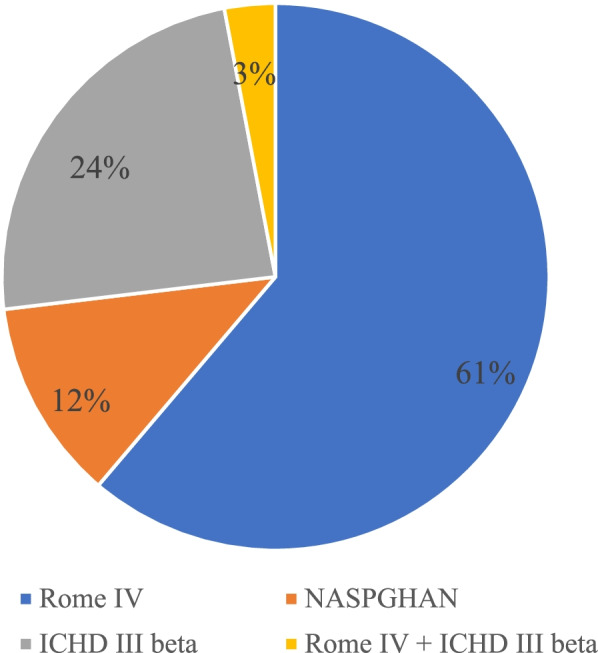
Cyclic vomiting syndrome diagnostic criteria generally adopted by respondents to the survey

Rome IV diagnostic criteria were used by 41 clinics (61.2%), the International Classification of Headache Disorders (ICHD)-III beta criteria by 16 clinics (23.9%), the North American Society for Pediatric Gastroenterology, Hepatology & Nutrition (NASPGHAN) criteria by 8 clinics (11.9%), while 2 clinics (3%) applied both Rome IV and ICHD-III beta criteria (Fig. 3). Table 1 illustrate the criteria adopted according to specific outpatient clinic. A statistically significant difference was found between the adopted diagnostic criteria and the type of outpatient service (*p* = 0.002).

**Table 1:** Type of diagnostic criteria adopted according to specific outpatient clinic

Outpatient Clinic	Rome IV	NASPGHAN	ICHD III beta	Rome IV + ICHD III beta
Gastroenterology, n (%)	33 (80.5)	3 (7.3)	4 (9.8)	1 (2.4)
Neurology, n (%)	3 (20)	2 (13.3)	9 (60)	1 (6.7)
Neuro-Gastroenterology, n (%)	4 (44.4)	2 (22.2)	3 (33.3)	0 (0)
CVS, n (%)	0 (0)	1 (100)	0 (0)	0 (0)
Headache, n (%)	1 (100)	0 (0)	0 (0)	0 (0)

*Abbreviations*: *CVS* cyclic vomiting syndrome

### Differential diagnosis

The main organic causes of vomiting identified in patients with suspected CVS were gastrointestinal diseases in 36 clinics (53.7%), neurologic diseases in 33 (49.2%), metabolic and endocrinologic causes in 19 (28.3%), infections in 19 (28.3%), urologic causes in 3 (4.5%) and other causes in 3 (4.5%), which included genetic causes in 2 (3%) and appendicitis in 1 (1.5%). Supplementary Table 2 summarizes the main organic causes identified by clinicians according to specific outpatient clinic. No statistical difference was identified among the different clinic settings excepting for gastrointestinal diseases, which were more commonly identified by gastroenterologists (*p* = 0.006). Among responders to the open-ended question (27, 40.3%), pathologies identified as cause of vomiting were: epilepsy in 8 patients (Panayiotopoulos syndrome in 5), appendicitis in 5, food intolerance in 4, intestinal obstruction in 5, coeliac disease in 3, congenital cytomegalovirus infection in 3, volvulus in 2, pseudotumor cerebri in 2, inflammatory bowel disease in 2, esophagitis in 2, brain tumor in 2, gastritis in 1, *Helicobacter Pylori* infection in 1, post enteritis syndrome in 1, ion channel disease in 1, superior mesenteric artery syndrome in 1, GLUT-1 deficiency in 1, malrotation in 1, Chiari malformation in 1.

### Time to diagnosis

The majority of units (42 clinics, 62.7%) received a referral for suspected CVS after 3 to 5 acute episodes, whilst almost one third of centers (22, 32.8%) used to receive the referrals after more than 5 acute attacks. Three responders (4.5%) declared to receive referrals for suspected CVS only after 2 vomiting episodes. No statistical difference was found between number of attacked recorded at the time of referral and type of outpatient clinic (*p* = 0.86, ns).

### Comorbidities

The most common comorbidities recorded were headache by 63 clinics (94%), anxiety by 34 (50.7%), sleep disorders by 29 (43.3%), irritable bowel syndrome by 28 (41.8%); other comorbidities were recorded by 6 clinics (9%), and in particular neurodevelopmental delay by 2 (3%), constipation by 1 (1.5%), coeliac disease by 1 (1.5%), vertigo by 1 (1.5%) and eating disorder by 1 (1.5%). No comorbidities were recorded by 1 clinic (1.5%). Comorbidities recorded among patients with CVS according to specific outpatient clinic are summarized in Supplementary Table 3. No statistical difference was identified among the different clinic settings. Among responders to the open-ended question (26 clinics, 38.8%), the main comorbidities identified in patients with CVS were: headache in 107 patients, irritable bowel syndrome in 65, sleep disturbance in 51, anxiety in 22, migraine in 9, parasomnia in 4, autism in 1, coeliac disease in 1, abdominal pain in 4, delayed sleep induction in 1, vertigo in 1.

### Family history

A family history of migraine was detected by 58 clinics (86.6%), functional GI disorders by 30 (44.8%), childhood periodic syndromes (including benign paroxysmal torticollis, benign paroxysmal vertigo, and abdominal migraine) by 20 (29.9%), cyclic vomiting syndrome by 10 (14.9%). Supplementary Table 4 depicts the family history of diseases detected among patients with CVS according to specific outpatient clinic. No statistical difference was identified among the different clinic settings excepting for childhood periodic syndromes (*p* = 0.046). Among responders to the open-ended question (22 clinics, 32.8%), a family history of migraine was identified in 206 patients, functional GI disorders in 37, CVS in 7, irritable bowel syndrome in 2, benign paroxysmal vertigo in 1.

### Triggers

Stress was the most common trigger encountered among patients and it was reported by 77.6% (52) of the responders, followed by sleep deprivation by 27 (40.3%), excessive excitement by 22 (32.8%), infections by 20 (30%), menstrual cycle by 9 (13.4%), physical exercise and foods by 6 (9%), and other factors by 3 (4.5%), including fever, sunlight exposure and car traveling. Supplementary Table 5 describes main triggers identified among patients according to specific outpatient clinic. No statistical difference was identified among the different clinic settings excepting for foods, who were more commonly identified by gastroenterologists and neuro-gastroenterologists (*p* = 0.019).

Among responders to the open-ended question (18 clinics, 26.9%), stress was a trigger factor in 105 patients, infection in 22, excessive excitement in 6, sleep deprivation 5 patients, menstrual cycle in 4.

### Screening tests

Almost all survey responders (63, 94%) performed baseline testing toward identifying organic causes, including full blood count, C-reactive protein, electrolytes, glycemia, creatinine, uremia, aspartate aminotransferase, alanine aminotransferase, gamma-glutamyl transferase, amylase, lipase and urine analysis; 48 respondents (71.6%) performed blood gas analysis; 45 (67.2%) coeliac screening; 35 (52.2%) ammonia and lactic acid levels; 25 (37.3%) upper GI series; 23 (34.3%) ultrasound of the abdomen and pelvis; 24 (35.8%) electroencephalogram (EEG); 16 (23.9%) brain magnetic resonance imaging; 5 (7.5%) upper GI endoscopy with biopsies; 2 (3%) other investigations, such as urinary organic acids and serum amino acids, ophthalmology consultation and child neuropsychiatry consultations. Two respondents (3%) did not perform any investigation. Supplementary Table 6 summarizes screening tests performed according to specific outpatient clinic. No statistical difference was identified among the different clinic settings excepting for ammonia and lactic acid levels, which were more commonly performed by gastroenterologists (*p* = 0.015).

### Treatment

During the prodromal phase, the most widely used drug was the ondansetron (35 clinics, 52.2%), followed by sedatives, such as lorazepam and midazolam (11 clinics, 16.4%), sumatriptan (8, 11.9%), and aprepitant (6, 9%). Other drugs, such as anticonvulsants, analgesics, vitamin B6, steroids, and a combination of indomethacin, caffeine and prochlorperazine were also used (6 clinics, 9%). Twenty-three (34.3%) responders did not use any drug during the prodromal phase.

Ondansetron was the most commonly used medication during the emetic phase (56 clinics, 83.6%), together with supportive cares, such as decrease stimulation in a dark, quiet, private room (41, 61.2%), intravenous (IV) infusion of 10% glucose (35, 52.2%), or IV infusion of saline solution (14, 20.9%). Proton pump inhibitors were used during emetic phase by 17 clinics (25.4%), H_2_-receptor antagonists by 5 (7.5%), sedatives by 17 (26.9%), non-steroidal anti-inflammatory analgesics by 7 (10.4%), and other treatments, such as paracetamol, steroids, and oral rehydration, by 7 (9%).

Lifestyle changes and reassurance were promoted for the interictal period by 43 units (64.2%). Prophylactic pharmacotherapy commonly used was cyproheptadine by 34 (50.7%), pizotifen by 17 (25.4%), amitriptyline by 17 (25.4%), mitochondrial supplements by 10 (14.9%), anticonvulsants by 7 (10.4%), aprepitant 4 (6%), propranolol 4 (6%). Other treatments used by 8 units (11.9%) included flunarizine, 5-hydroxytryptophan, proton pump inhibitors, paracetamol, magnesium supplementation.

Supplementary Table 7 summarizes treatments applied in patients with CVS according to specific outpatient clinic. No statistical difference was encountered among the different clinic settings excepting for H_2_-receptor antagonists (*p* = 0.004) and sedatives (*p* = 0.03).

### Outcomes

Regarding the most common long-term outcomes, progression to migraine was reported by 25 (37.3%), the persistence of symptoms with prolonged well-being phase by 21 responders (31.3%), complete resolution by 20 (29.9%), progression to other functional GI disorders by 6 (9%), and persistence of symptoms with the same characteristics by 4 (6%). Supplementary Table 8 depicts the long-term outcomes identified according to specific outpatient clinic. In particular, significant difference was reported among clinics regarding progression to migraine (*p* < 0.001) and persistence of symptoms with prolonged well-being phase (*p* = 0.024). No statistical correlation was found between the family history of disease or prophylactic therapy preferentially adopted and long-term outcomes.

### Study limitations

Although to our knowledge the present study represents the most extensive data collection on current national practice concerning the diagnosis and management of patients with CVS, we have to acknowledge some limitations: the survey was designed to be rapid and easy to complete to involve a large number of centers, although the collected data lacked in precision; therefore, it does not guarantee the reliability of the results. Moreover, despite the large adhesion, we cannot be sure that all the centers that care for patients with CVS responded to the questionnaire. However, the aforementioned wide geographical distribution and the high response rate is likely to be a reliable national representation.

## Discussion

This web-based national survey involving all members of the SIGENP and SINP evaluate attitudes and practices of Italian tertiary care Centers regarding the management of children with CVS. The geographical distribution (16/20 regions) and the high response rate suggest that the present data collection might be a reliable representative picture. Most of the respondent units cared for less than 20 patients (88%). Moreover, more than half of the patients were referred after 3 to 5 episodes, and a quarter after 5 attacks. Considering the difficulty in distinguishing CVS from other organic pathologies, a multidisciplinary team approach is desirable to detect minor abnormalities and guide the baseline screening investigations. However, only a limited number of combined neuro-gastroenterology outpatient clinic and only 1 specialized CVS clinic are available in Italy, and patients were mainly followed-up by either pediatric gastroenterologists or neurologists alone.

Interestingly, the diagnostic approach recorded was different among units and the relationship between diagnostic criteria adopted and the type of outpatient service resulted statistically significant in our report. In particular, Rome IV criteria [2, 3] were adopted by 80.5% of gastroenterologist, while only 20% of neurologist; contrariwise, ICHD-III beta criteria [19] were used by 60% of neurologists and 10% of gastroenterologists. Although widely used, many differences exist among these criteria: the peculiarity of the Rome IV criteria [2, 3] is the higher sensitivity at the expense of a lower specificity, as only two typical episodes are required to formulate the diagnosis, while at least five for both ICHD-III beta [19] and NASPGHAN criteria [1]. Moreover, the former accepts the possibility of some mild inter-episodic symptoms, such as irritable bowel syndrome, nausea, and dyspepsia. Therefore, the type of diagnostic criteria adopted might affect the diagnostic process.

Differently from other functional GI disorders, CVS should not be diagnosed based solely on diagnostic criteria as a careful assessment of symptoms is needed and many organic causes should be ruled out. In facts, several multidisciplinary organic diseases emerged from our survey, highlighting a critical point often emphasized in literature regarding the vast differential diagnosis that should be examined in patients with CVS, which might contribute to the diagnostic delay [1, 2, 20–23]. Thereby, currently available consensus guidelines [1] recommend a baseline screening including routine blood tests and upper GI series, although other investigations are fostered in the presence of specific alarm features, such as abdominal signs (i.e., bilious vomiting), abnormal neurological examination (i.e., papilledema, confusion), triggering factors (i.e., high fat or protein meal, prolonged fasting) and a changing pattern or worsening of vomiting episodes.

Nevertheless, we report different diagnostic approaches among Italian clinicians, which was particularly evident when comparing gastroenterologists and neurologists, with the former more prone in prescribing metabolic tests, such as ammonia and lactic acid, while the latter EEG tests. Interestingly, significant higher rates of gastrointestinal illness were identified among patients with suspected CVS by gastroenterologists, compared to other clinicians. Two units stated to perform no investigations test before the diagnosis, which underlies the need for more widespread and uniform information about the diagnostic process.

Almost all the responders recognized headache as frequent comorbidity in patients with CVS, and three quarters reported a family history of migraine; this endorsing the link between CVS and migraine [24]. In fact, several analogies have been recognized between the two, concerning demographic, trigger, and relieving factors and associated clinical features [25]. Moreover, two mitochondrial polymorphisms were found to be highly associated with both conditions [26]. Potential common pathophysiology in CVS and migraine was recently demonstrated by Ellingsen et colleagues [27], who firstly reported altered insular connectivity with the sensorimotor network at functional magnetic resonance imaging in both the pathologies compared to healthy controls. Another common comorbidity recorded was anxiety, which was reported by half of the responders. Analogously to other functional GI disorders [28, 29], the risk of anxiety is increased in children with CVS [30], and it seems to affect the quality of life more than the disease characteristics do, such as frequency and duration of the attacks [31]. Noteworthy, improvement of CVS symptoms was reported in a child using cognitive behavioral therapy and heart rate variability biofeedback, pointing out the potential clinical impact of screening for psychiatric symptoms as part of the medical evaluation of children with CVS [32]. This aspect is particularly relevant, as stress can trigger CVS episodes [33]. Our report distinguished the negative stress from the excitement, and 78% of respondents recognized the former as a common trigger, while only 31% recognized the latter. Sleep deprivation was another common trigger (43%), hence proper sleep hygiene should also be emphasized.

Our survey confirmed the wide variety of therapeutic strategies used in patients with CVS. In the absence of large clinical trials, optimal treatment remains undetermined, and therapeutic indications are mostly driven in pediatrics by the NASPGHAN consensus guidelines, mainly based on expert opinion [1]. Because treatment responses are variable, it is plausible that clinicians often have to navigate between different medications [34]. However, our survey demonstrated a predilection of certain drugs during emetic phase according to specific clinic, which reflects the cultural background of physicians (gastroenterologists vs neurologists). No significant differences were observed regarding the management approach during the other phases of the illness. Ondansetron was the most prescribed medication during prodromal phase. Strong evidence is available regarding the effectiveness of ondansetron in terminating the vomiting episodes [13, 30, 35, 36], but its role in the prodromal phase needs further evaluation [37].

In line with literature data [12], our study demonstrated that the natural history of CVS is largely variable: neurologists reported significantly more frequently a progression to migraine, while gastroenterologists a persistence of symptoms with prolonged wellbeing phase; only a minority of the responders (7.5%) reported persistence of symptoms with unvaried features.

## Conclusions

In conclusion, our survey highlights poor consensus amongst clinicians in our country in the diagnosis and the management of children with CVS. Although this survey reports a generally appropriate referral timing to tertiary level centers, the small number of patients followed-up per center compared to that from other countries supports the need for a wider dissemination among Italian pediatricians, because CVS is probably an under recognized condition that, when considered, is not hard to diagnose. There was no uniform opinion concerning the diagnostic and therapeutic algorithm, raising the need for a national consensus guideline in order to standardize the practice. A multicenter prospective study evaluating medical history, clinical features, laboratory and instrumental tests and their possible correlation with response to treatment and long-term outcomes might help identify specific disease phenotypes that might direct the diagnosis and therapy in children with CVS.

## Supplementary Information

**Additional file 1: Supplementary Table 1.** Web-based Questionnaire [original language].

**Additional file 2: Supplementary Table 2.** Main organic causes of vomiting identified in patients with suspected cyclic vomiting syndrome according to specific outpatient clinic.

**Additional file 3: Supplementary Table 3.** Comorbidities recorded among patients with cyclic vomiting syndrome according to specific outpatient clinic.

**Additional file 4: Supplementary Table 4.** Family history of diseases detected among patients with cyclic vomiting syndrome according to specific outpatient clinic.

**Additional file 5: Supplementary Table 5**. Triggers recorded among patients with cyclic vomiting syndrome according to specific outpatient clinic.

**Additional file 6: Supplementary Table 6.** Screening tests performed among patients with cyclic vomiting syndrome according to specific outpatient clinic.

**Additional file 7: Supplementary Table 7.** Treatments applied in patients with cyclic vomiting syndrome according to specific outpatient clinic.

**Additional file 8: Supplementary Table 8.** Long-term outcomes identified among patients with cyclic vomiting syndrome according to specific outpatient clinic.

## Data Availability

All data generated or analyzed during this study are included in this published article.

## Abbreviations

CVS: Cyclic Vomiting Syndrome
EEG: Electroencephalogram
GI: Gastrointestinal
IV: Intravenous
SIGENP: Italian Society of Pediatric Gastroenterology, Hepatology and Nutrition
SINP: Italian Society of Pediatric Neurology
LR: Likelihood Ratio test
