# Supplementary material for: Cyclic vomiting syndrome in children: a nationwide survey of current practice on behalf of the Italian Society of Pediatric Gastroenterology, Hepatology and Nutrition (SIGENP) and Italian Society of Pediatric Neurology (SINP)
Source: Ital J Pediatr. 2022 Aug 30;48:156. doi: 10.1186/s13052-022-01346-y (PMC9429644; doi:10.1186/s13052-022-01346-y)
Supplement: Supplementary file 1 — Additional file 1: Supplementary Table 1. Web-based Questionnaire [original language]. [file 13052_2022_1346_MOESM1_ESM.docx]

**Supplementary Table 1.** Web-based Questionnaire [original language]

1. Denominazione completa della struttura dove lavori (Nome Ospedale, Città, Nome UO):

...........................................................

1. Quanti pazienti con vomito ciclico hai/avete in cura presso la tua/vostra struttura?
   1. Meno di 10
   2. Tra 10 e 15
   3. Tra 15 e 20
   4. altro .................. (specificare)

2.1) Se possibile, specificare il numero di pazienti

……………………………………………………………………………

1. Nella struttura dove lavori esiste un ambulatorio dedicato alla diagnosi e cura dei pazienti con vomito ciclico?
   1. Ambulatorio di pediatria generale
   2. Ambulatorio di gastroenterologia pediatrica
   3. Ambulatorio di neurologia pediatrica
   4. Ambulatorio congiunto gastroenterologia/neurologia
   5. Ambulatorio dedicato a pazienti con vomito ciclico
   6. Altro .................(specificare)
2. A quali criteri diagnostici fai riferimento per la diagnosi di vomito ciclico?
   1. NASPGHAN 2008
   2. Roma IV 2016
   3. The International Classification of Headache Disorders (ICHD III)
   4. Altro ....................(specificare)
3. Quale patologia ti è capitato di diagnosticare come causa secondaria di un vomito ricorrente? (si può dare più di una risposta, specificare quale patologia e quanti casi)
   1. Cause gastrointestinali (malformazioni apparato digerente, infiammatorie, malassorbimento, pancreatiche)
   2. Cause neurologiche (tumori encefalo, epilessia autonomica/S. di panayiotopoulos, idrocefalo, ematoma subdurale)
   3. Cause metaboliche ed endocrinologiche
   4. Cause urologiche (ostruzione pielo-ureterale, nefrolitiasi, etc.)
   5. Cause infettive (enteriti, otite media, sinusite cronica, epatite)
   6. Altro .......................... (specificare)

5.1) Se possibile, specificare le patologie e il numero di casi

…………………………………………………………………………………………..

1. Dopo quanti episodi di vomito ricorrente il paziente è stato inviato presso la tua struttura nel sospetto di vomito ciclico?
   1. 2 episodi
   2. Tra 3 e 5 episodi
   3. Più di 5 episodi
   4. Altro ....................(specificare)
2. Quali comorbidità hai riscontrato nella tua casistica? (si può dare più di una risposta)
   1. Ansia
   2. cefalea
   3. Intestino irritabile
   4. Disturbi del sonno
   5. Nessuna
   6. altro .....................(specificare)

7.1) Se possibile, specificare tipo di comorbidità e numero

………………………………………………………………………………

1. I pazienti che hai in cura per vomito ciclico hanno familiarità per una di queste patologie? (si può dare più di una risposta)
   1. Vomito ciclico
   2. Emicrania
   3. Disordini funzionali gastrointestinali
   4. Sindromi periodiche familiari (vertigine parossistica benigna della infanzia)
   5. Altro .....................(specificare)

8.1) Se possibile specificare il tipo di patologia e il numero

………………………………………………………………………………………

1. Quali trigger riscontri nella tua casistica? (si può dare più di una risposta)
   1. stress
   2. eccitazione (matrimonio, compleanno, vacanze)
   3. deprivazione del sonno
   4. esercizio fisico
   5. infezioni
   6. ciclo mestruale
   7. Alcuni cibi (cibi ricchi di glutammato monosodico, ricchi di coloranti, caffeina)
   8. Altro ................................ (specificare)

9.1) Se possibile, specificare il tipo di trigger e il numero di casi

………………………………………………………….

1. Quale è l’outcome a lungo termine nella tua popolazione di pazienti?
   1. Risoluzione
   2. progressione verso l’emicrania
   3. progressione verso un Disordine Funzionale Gastrointestinale
   4. persistenza con intervalli prolungati di benessere
   5. persistenza della malattia con caratteristiche simili
   6. Altro ......................(specificare)

10.1) Se possibile specificare il tipo di outcome e il numero di casi

…………………………………………………………………

1. Quali sono gli esami di prima linea che effettui nel bambino con vomito ciclico? (si può inserire più di una risposta)
   1. Nessuno
   2. Emocromo, PCR, elettroliti, glicemia, creatinina, azotemia, AST, ALT, GGT, amilasi, lipasi, esame urine
   3. Equilibrio acido base (Emogas)
   4. ammoniemia, lattacidemia
   5. sierologia per celiachia
   6. Rx digerente prime vie con m.d.c.
   7. EGDS con biopsie
   8. Ecografia addome
   9. RM encefalo
   10. EEG
   11. Altro ..................(specificare)
2. Quali farmaci utilizzi nella fase prodromica? (si può inserire più di una risposta)
   1. Ondansetron
   2. Sumatriptan
   3. Aprepitant
   4. Sedativi (Lorazepam, Midazolam)
   5. Nessuno
   6. Altro ................(specificare)
3. Quali strategie utilizzi abitualmente nella fase emetica? (si può inserire più di una risposta)
   1. Stanza buia e tranquilla
   2. Ondansetron
   3. Infusione ev di fisiologica
   4. Infusione ev di glucosata al 10% con elettroliti
   5. H2-antagonisti
   6. Inibitori di pompa protonica
   7. Sedativi (midazolam)
   8. Antidolorifici (chetorolac)
   9. Altro ..........................(specificare)
4. Quali strategie utilizzi abitualmente per la profilassi? (si può inserire più di una risposta)
   1. Modifiche dello stile di vita
   2. ciproeptadina
   3. pizotifene
   4. propanololo
   5. amitriptilina
   6. aprepitant
   7. Antiepilettici (es. topiramato, fenobarbitale, ac. valproico)
   8. Supplementazione mitocondriale (L-carnitina, coenzima Q10)
   9. Altro ....................(specificare)

14.1) Se possibile, specificare il tipo di profilassi ed il numero di pazienti attualmente in terapia

………………………………………………………………………….
