# Supplementary material for: Cyclic vomiting syndrome in children: a nationwide survey of current practice on behalf of the Italian Society of Pediatric Gastroenterology, Hepatology and Nutrition (SIGENP) and Italian Society of Pediatric Neurology (SINP)
Source: Ital J Pediatr. 2022 Aug 30;48:156. doi: 10.1186/s13052-022-01346-y (PMC9429644; doi:10.1186/s13052-022-01346-y)
Supplement: Supplementary file 2 — Additional file 2: Supplementary Table 2. Main organic causes of vomiting identified in patients with suspected cyclic vomiting syndrome according to specific outpatient clinic. [file 13052_2022_1346_MOESM2_ESM.docx]

**Supplementary Table 2.** Main organic causes of vomiting identified in patients with suspected cyclic vomiting syndrome according to specific outpatient clinic.

| Organic causes | Gs,  n (%) | Neurology,  n (%) | Neuro-Gs,  n (%) | CVS,  n (%) | Headache,  n (%) | p-value |
| --- | --- | --- | --- | --- | --- | --- |
| GI | 21 (31.3) | 5 (7.5) | 9 (13.4) | 1 (1.5) | 0 (0) | **0.006** |
| Neurologic | 18 (26.9) | 9 (13.4) | 6 (9) | 0 (0) | 0 (0) | 0.334 |
| Metabolic and endocrinologic | 13 (19.4) | 3 (4.5) | 3 (4.5) | 0 (0) | 0 (0) | 0.666 |
| Infectious | 7 (10.4) | 0 (0) | 3 (4.5) | 0 (0) | 1 (1.5) | 0.072 |
| Urologic | 2 (3) | 0 (0) | 1 (1.5) | 0 (0) | 0 (0) | 0.45 |
| Other | 1 (1.5) | 1 (1.5) | 1 (1.5) | 0 (0) | 0 (0) | 0.485 |

Abbreviations: GI, gastrointestinal, Gs, gastroenterology.
