# Supplementary material for: Cyclic vomiting syndrome in children: a nationwide survey of current practice on behalf of the Italian Society of Pediatric Gastroenterology, Hepatology and Nutrition (SIGENP) and Italian Society of Pediatric Neurology (SINP)
Source: Ital J Pediatr. 2022 Aug 30;48:156. doi: 10.1186/s13052-022-01346-y (PMC9429644; doi:10.1186/s13052-022-01346-y)
Supplement: Supplementary file 3 — Additional file 3: Supplementary Table 3. Comorbidities recorded among patients with cyclic vomiting syndrome according to specific outpatient clinic. [file 13052_2022_1346_MOESM3_ESM.docx]

**Supplementary Table 3.** Comorbidities recorded among patients with cyclic vomiting syndrome according to specific outpatient clinic.

| Comorbidities | Gs,  n (%) | Neurology,  n (%) | Neuro-Gs,  n (%) | CVS,  n (%) | Headache,  n (%) | p-value |
| --- | --- | --- | --- | --- | --- | --- |
| Headache | 37 (55.2) | 15 (22.4) | 9 (13.4) | 1 (1.5) | 1 (1.5) | 0.287 |
| Anxiety | 23 (34.3) | 4 (6) | 6 (9) | 1 (1.5) | 0 (0) | 0.088 |
| IBS | 18 (26.9) | 5 (7.5) | 4 (6) | 1 (1.5) | 0 (0) | 0.763 |
| Sleep disorders | 13 (19.4) | 10 (14.9) | 4 (6) | 1 (1.5) | 1 (1.5) | 0.062 |
| Other | 3 (4.5) | 2 (3) | 1 (1.5) | 0 (0) | 0 (0) | 0.772 |

Abbreviations: Gs, gastroenterology, IBS, irritable bowel syndrome
