# Supplementary material for: Cyclic vomiting syndrome in children: a nationwide survey of current practice on behalf of the Italian Society of Pediatric Gastroenterology, Hepatology and Nutrition (SIGENP) and Italian Society of Pediatric Neurology (SINP)
Source: Ital J Pediatr. 2022 Aug 30;48:156. doi: 10.1186/s13052-022-01346-y (PMC9429644; doi:10.1186/s13052-022-01346-y)
Supplement: Supplementary file 4 — Additional file 4: Supplementary Table 4. Family history of diseases detected among patients with cyclic vomiting syndrome according to specific outpatient clinic. [file 13052_2022_1346_MOESM4_ESM.docx]

**Supplementary Table 4.** Family history of diseases detected among patients with cyclic vomiting syndrome according to specific outpatient clinic.

| Family history | Gs,  n (%) | Neurology,  n (%) | Neuro-Gs,  n (%) | CVS,  n (%) | Headache,  n (%) | p-value |
| --- | --- | --- | --- | --- | --- | --- |
| Migraine | 34 (50.7) | 13 (19.4) | 9 (13.4) | 1 (1.5) | 1 (1.5) | 0.405 |
| Functional GI disorders | 22 (32.8) | 4 (6) | 3 (4.5) | 1 (1.5) | 0 (0) | 0.151 |
| Childhood periodic syndromes | 8 (12) | 8 (12) | 3 (4.5) | 0 (0) | 1 (1.5) | **0.046** |
| CVS | 6 (9) | 3 (4.5) | 1 (1.5) | 0 (0) | 0 (0) | 0.823 |

Abbreviations: Gs, gastroenterology, GI, gastrointestinal, CVS, cyclic vomiting syndrome
