# Supplementary material for: Cyclic vomiting syndrome in children: a nationwide survey of current practice on behalf of the Italian Society of Pediatric Gastroenterology, Hepatology and Nutrition (SIGENP) and Italian Society of Pediatric Neurology (SINP)
Source: Ital J Pediatr. 2022 Aug 30;48:156. doi: 10.1186/s13052-022-01346-y (PMC9429644; doi:10.1186/s13052-022-01346-y)
Supplement: Supplementary file 5 — Additional file 5: Supplementary Table 5. Triggers recorded among patients with cyclic vomiting syndrome according to specific outpatient clinic. [file 13052_2022_1346_MOESM5_ESM.docx]

**Supplementary Table 5.** Triggers recorded among patients with cyclic vomiting syndrome according to specific outpatient clinic.

| Triggers | Gs,  n (%) | Neurology,  n (%) | Neuro-Gs,  n (%) | CVS,  n (%) | Headache,  n (%) | p-value |
| --- | --- | --- | --- | --- | --- | --- |
| Stress | 30 (44.8) | 12 (17.9) | 8 (11.9) | 1 (1.5) | 1 (1.5) | 0.832 |
| Sleep deprivation | 14 (20.9) | 4 (6) | 8 (11.9%) | 1 (1.5) | 0 (0) | 0.413 |
| Excessive excitement | 13 (19.4) | 6 (9) | 2 (3) | 1 (1.5) | 0 (0) | 0.66 |
| Infections | 14 (20.9) | 2 (3) | 2 (3) | 1 (1.5) | 1 (1.5) | 0.282 |
| Menstrual cycle | 7 (10.4) | 1 (1.5) | 1 (1.5) | 0 (0) | 0 (0) | 0.588 |
| Physical exercise | 5 (7.5) | 0 (0) | 0 (0) | 0 (0) | 1 (1.5) | 0.205 |
| Foods | 3 (4.5) | 0 (0) | 3 (4.5) | 0 (0) | 0 (0) | **0.019** |
| Other | 1 (1.5) | 1 (1.5) | 1 (1.5) | 0 (0) | 0 (0) | 0.485 |

Abbreviations: Gs, gastroenterology.
