# Supplementary material for: Cyclic vomiting syndrome in children: a nationwide survey of current practice on behalf of the Italian Society of Pediatric Gastroenterology, Hepatology and Nutrition (SIGENP) and Italian Society of Pediatric Neurology (SINP)
Source: Ital J Pediatr. 2022 Aug 30;48:156. doi: 10.1186/s13052-022-01346-y (PMC9429644; doi:10.1186/s13052-022-01346-y)
Supplement: Supplementary file 6 — Additional file 6: Supplementary Table 6. Screening tests performed among patients with cyclic vomiting syndrome according to specific outpatient clinic. [file 13052_2022_1346_MOESM6_ESM.docx]

**Supplementary Table 6.** Screening tests performed among patients with cyclic vomiting syndrome according to specific outpatient clinic.

| Screening tests | Gs,  n (%) | Neurology,  n (%) | Neuro-Gs,  n (%) | CVS,  n (%) | Headache,  n (%) | p-value |
| --- | --- | --- | --- | --- | --- | --- |
| BBT | 39 (58.2) | 14 (20.9) | 8 (11.9) | 1 (1.5) | 1 (1.5) | 0.777 |
| BGA | 31 (46.3) | 10 (14.9) | 5 (7.5) | 1 (1.5) | 1 (1.5) | 0.451 |
| Coeliac screening | 31 (46.3) | 8 (12) | 4 (6) | 1 (1.5) | 1 (1.5) | 0.099 |
| Ammonia and lactic acid levels | 26 (38.8) | 3 (4.5) | 5 (7.5) | 1 (1.5) | 0 (0) | **0.015** |
| Upper GI series | 18 (26.9) | 2 (3) | 4 (6) | 1 (1.5) | 0 (0) | 0.097 |
| Abdomen and pelvis US | 13 (19.4) | 5 (7.5) | 4 (6) | 1 (1.5) | 0 (0) | 0.765 |
| EEG | 11 (16.4) | 9 (13.4) | 3 (4.5) | 0 (0) | 1 (1.5) | 0.071 |
| UGE | 5 (7.5) | 0 (0) | 0 (0) | 0 (0) | 0 (0) | 0.205 |
| Other | 2 (3) | 0 (0) | 0 (0) | 0 (0) | 0 (0) | 0.547 |

Abbreviations: Gs, gastroenterology, BBT, baseline blood testing; BGA, blood gas analysis, US, ultrasound; EEG, electroencephalogram, UGE, upper gastrointestinal endoscopy
