# Supplementary material for: Cyclic vomiting syndrome in children: a nationwide survey of current practice on behalf of the Italian Society of Pediatric Gastroenterology, Hepatology and Nutrition (SIGENP) and Italian Society of Pediatric Neurology (SINP)
Source: Ital J Pediatr. 2022 Aug 30;48:156. doi: 10.1186/s13052-022-01346-y (PMC9429644; doi:10.1186/s13052-022-01346-y)
Supplement: Supplementary file 7 — Additional file 7: Supplementary Table 7. Treatments applied in patients with cyclic vomiting syndrome according to specific outpatient clinic. [file 13052_2022_1346_MOESM7_ESM.docx]

**Supplementary Table 7.** Treatments applied in patients with cyclic vomiting syndrome according to specific outpatient clinic.

| Treatments | Gs,  n (%) | Neurology,  n (%) | Neuro-Gs,  n (%) | CVS,  n (%) | Headache,  n (%) | p-value |
| --- | --- | --- | --- | --- | --- | --- |
| Prodromal phase  Ondansetron  Sedatives  Sumatriptan  Aprepitant  Other  None | 22 (32.8)  6 (9)  6 (9)  4 (6)  2 (3)  13 (19.4) | 7 (10.4)  4 (6)  1 (1.5)  1 (1.5)  2 (3)  5 (7.5) | 4 (6)  0 (0)  0 (0)  0 (0)  1 (1.5)  5 (7.5) | 1 (1.5)  1 (1.5)  1 (1.5)  1 (1.5)  0 (0)  0 (0) | 1 (1.5)  0 (0)  0 (0)  0 (0)  1 (1.5)  0 (0) | 0.826  0.21  0.37  0.601  0.528  0.392 |
| Emetic phase  Ondansetron  Supportive cares  IV 10% glucose IV saline  PPI  H2 antagonists  Sedatives  NSAIDs  Other | 34 (50.7)  28 (41.8)  23 (34.3)  8 (11.9)  14 (20.9)  4 (6)  9 (13.4)  3 (4.5)  3 (4.5) | 12 (17.9)  7 (10.4)  7 (10.4)  4 (6)  1 (1.5)  0 (0)  7 (10.4)  2 (3)  3 (4.5) | 8 (11.9)  6 (9)  4 (6)  2 (3)  1 (1.5)  1 (1.5)  0 (0)  2 (3)  1 (1.5) | 1 (1.5)  0 (0)  1 (1.5)  0 (0)  1 (1.5)  0 (0)  0 (0)  0 (0)  0 (0) | 1 (1.5)  0 (0)  0 (0)  0 (0)  0 (0)  0 (0)  1 (1.5)  0 (0)  0 (0) | 0.853  0.323  0.323  0.846  0.064  **0.004**  **0.03**  0.399  0.399 |
| Interictal period  Lifestyle changes and reassurance  Cyproheptadine  Pizotifen  Amitriptyline  Mitochondrial sp  Anticonvulsants  Aprepitant  Propranolol  Other | 30 (44.8)  23 (34.3)  9 (13.4)  10 (14.9)  4 (6)  3 (4.5)  2 (3)  4 (6)  2 (3) | 9 (13.4)  4 (6)  6 (9)  4 (6)  4 (6)  3 (4.5)  1 (1.5)  0 (0)  4 (6) | 4 (6)  6 (9)  1 (1.5)  2 (3)  1 (1.5)  1 (1.5)  0 (0)  0 (0)  2 (3) | 0 (0)  1 (1.5)  1 (1.5)  1 (1.5)  0 (0)  0 (0)  1 (1.5)  0 (0)  0 (0) | 0 (0)  0 (0)  1 (1.5)  0 (0)  0 (0)  1 (1.5)  0 (0)  0 (0)  0 (0) | 0.181  0.088  0.263  0.969  0.259  0.399  0.747  0.302  0.056 |

Abbreviations: Gs, gastroenterology, PPI, proton pump inhibitors, H2-receptor antagonists, histamine type 2 receptor antagonists; NSAIDs, non-steroidal anti-inflammatory drugs; sp, supplements
