# Supplementary material for: Cyclic vomiting syndrome in children: a nationwide survey of current practice on behalf of the Italian Society of Pediatric Gastroenterology, Hepatology and Nutrition (SIGENP) and Italian Society of Pediatric Neurology (SINP)
Source: Ital J Pediatr. 2022 Aug 30;48:156. doi: 10.1186/s13052-022-01346-y (PMC9429644; doi:10.1186/s13052-022-01346-y)
Supplement: Supplementary file 8 — Additional file 8: Supplementary Table 8. Long-term outcomes identified among patients with cyclic vomiting syndrome according to specific outpatient clinic. [file 13052_2022_1346_MOESM8_ESM.docx]

**Supplementary Table 8.** Long-term outcomes identified among patients with cyclic vomiting syndrome according to specific outpatient clinic.

| Outcomes | Gs,  n (%) | Neurology,  n (%) | Neuro-Gs,  n (%) | CVS,  n (%) | Headache,  n (%) | p-value |
| --- | --- | --- | --- | --- | --- | --- |
| Migraine | 7 (10.4) | 13 (19.4) | 5 (7.5) | 0 (0) | 0 (0) | **<0.001** |
| Prolonged well-being phase | 18 (26.9) | 1 (1.5) | 2 (3) | 0 (0) | 0 (0) | **0.024** |
| Resolution | 12 (17.9) | 5 (7.5) | 1 (1.5) | 1 (1.5) | 1 (1.5) | 0.467 |
| Other FGIDs | 5 (7.5) | 1 (1.5) | 0 (0) | 0 (0) | 0 (0) | 0.481 |
| Unchanged | 2 (3) | 0 (0) | 2 (3) | 0 (0) | 0 (0) | 0.077 |

Abbreviations: GS, gastroenterology, FGIDs, functional gastrointestinal disorders
